# Frequency-specific corticofugal modulation of the dorsal cochlear nucleus in mice

**DOI:** 10.3389/fnsys.2014.00125

**Published:** 2014-07-01

**Authors:** Lingzhi Kong, Colin Xiong, Liang Li, Jun Yan

**Affiliations:** ^1^Department of Physiology and Pharmacology, Faculty of Medicine, Hotchkiss Brain Institute, University of CalgaryCalgary, AB, Canada; ^2^Department of Psychology, Department of Machine Intelligence, Speech and Hearing Research Center, Key Laboratory on Machine Perception (Ministry of Education), PKU-IDG/McGovern Institute for Brain Research, Peking UniversityBeijing, China

**Keywords:** corticofugal modulation, primary auditory cortex, dorsal cochlear nucleus, frequency-specific modulation, lemniscal, non-lemniscal, neural plasticity

## Abstract

The primary auditory cortex (AI) modulates the sound information processing in the lemniscal subcortical nuclei, including the anteroventral cochlear nucleus (AVCN), in a frequency-specific manner. The dorsal cochlear nucleus (DCN) is a non-lemniscal subcortical nucleus but it is tonotopically organized like the AVCN. However, it remains unclear how the AI modulates the sound information processing in the DCN. This study examined the impact of focal electrical stimulation of AI on the auditory responses of the DCN neurons in mice. We found that the electrical stimulation induced significant changes in the best frequency (BF) of DCN neurons. The changes in the BFs were highly specific to the BF differences between the stimulated AI neurons and the recorded DCN neurons. The DCN BFs shifted higher when the AI BFs were higher than the DCN BFs and the DCN BFs shifted lower when the AI BFs were lower than the DCN BFs. The DCN BFs showed no change when the AI and DCN BFs were similar. Moreover, the BF shifts were linearly correlated to the BF differences. Thus, our data suggest that corticofugal modulation of the DCN is also highly specific to frequency information, similar to the corticofugal modulation of the AVCN. The frequency-specificity of corticofugal modulation does not appear limited to the lemniscal ascending pathway.

## Introduction

Understanding the central processing of auditory information is incomplete without considering the descending systems. Morphologically, the auditory cortex sends a large number of descending fibers to the subcortical nuclei (corticofugal projections, Doucet et al., 2002, 2003; Coomes and Schofield, 2004), including the auditory thalamus (Roger and Arnault, 1989; Winer et al., 2001), inferior colliculus (Andersen et al., 1980; Faye-Lund, 1985; Coleman and Clerici, 1987; Herbert et al., 1991; Saldaña et al., 1996; Winer et al., 1998, 2002; Bajo and Moore, 2005; Coomes et al., 2005; Bajo et al., 2007; Peterson and Schofield, 2007; Markovitz et al., 2013) and cochlear nucleus (Weedman and Ryugo, 1996a,b; Jacomme et al., 2003; Schofield and Coomes, 2005a,b; Meltzer and Ryugo, 2006; Schofield et al., 2006). In addition, the corticofugal system implements a highly selective modulation of the physiological response in the subcortical nuclei (Yan and Suga, 1996, 1999; Ma and Suga, 2001a; Nakamoto et al., 2008; Bajo et al., 2010). In the frequency domain, focal activation of the primary auditory cortex (AI) shifts the receptive fields of neurons in the subcortical nuclei towards the best frequency (BF) of activated AI neurons and reorganizes the frequency maps of those subcortical nuclei including the ventral division of the medial geniculate body (MGBv; Zhang and Suga, 2000; Tang et al., 2012), the central nucleus of the inferior colliculus (ICc; Yan and Suga, 1998; Zhang and Suga, 2000; Yan et al., 2005; Yan and Ehret, 2001, 2002; Ma and Suga, 2001b, 2003) and even the anteroventral cochlear nucleus (AVCN; Luo et al., 2008; Liu et al., 2010).

Frequency-specific corticofugal modulation appears to be a feature of the lemniscal auditory pathway. Up to now, it has been exclusively observed in the lemniscal subcortical nuclei (MGBv, ICc and AVCN) and not in the non-lemniscal nuclei (Calford and Aitkin, 1983; Imig and Morel, 1983; Hu et al., 1994) including those found in the medial division of the medial geniculate body (MGBm) and the external nucleus of the inferior colliculus (ICx; Jen et al., 2001; Zhang and Suga, 2005; Wu and Yan, 2007; Tang et al., 2012). Given that the lemniscal auditory pathway is also characterized by a sharp tuning in sound frequency and a strict tonotopic projection (Calford, 1983; Rodrigues-Dageff et al., 1989; Redies and Brandner, 1991; Anderson and Linden, 2011), the question raised here is whether the frequency-specificity of corticofugal modulation is limited to the lemniscal system or dominated by the tonotopy regime of the central auditory system.

The dorsal cochlear nucleus (DCN) is tonotopically organized (Young et al., 1992; Luo et al., 2009). In contrast to the AVCN, the DCN receives inputs from both the auditory and somatosensory systems (Baizer et al., 2012), and projects to the ICx and MGBm (Malmierca et al., 2002). The DCN could therefore be a non-lemniscal nucleus because it is tightly associated to non-lemniscal auditory system (Malmierca et al., 2002; Ryugo et al., 2003; Luo et al., 2012). In the present study, we examined the effects of focal electrical stimulation of the AI (ES_AI_) on the auditory responses of the DCN neurons. Our data show that ES_AI_ induced a frequency-specific shift in the frequency tunings of the DCN neurons, similar to the modulation of the AVCN (Luo et al., 2008; Liu et al., 2010).

## Materials and methods

**Figure 1:**
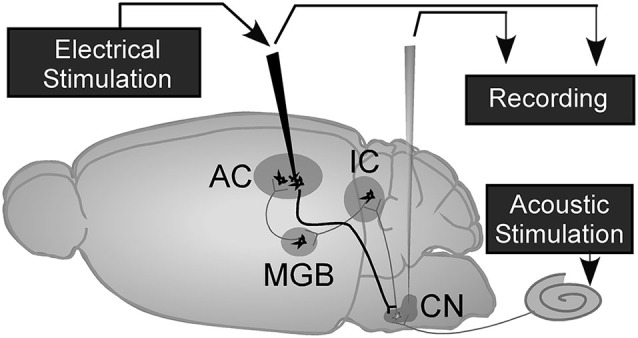
**A schematic of the mouse brain with recording and electrical stimulation sites (adapted from Luo et al., 2008)**. The corticofugal projection to DCN is indicated by a solid dark line. Auditory cortex, AC; medial geniculate body, MGB; inferior colliculus, IC; cochlear nucleus, CN.

C57 female mice, aged 4–7 weeks and weighing 14.6–20.7 g, were used in this study. All protocols and procedures were approved by the Animal Care Committee of the University of Calgary (protocol number: M04044). Animals were anesthetized with a mixture of ketamine (85 mg/kg, i.p., Bimeda-MTC Animal Health Inc., Canada) and xylazine (15 mg/kg, i.p., Bimeda-MTC Animal Health Inc.). The anesthetic level was maintained by additional doses of ketamine and xylazine, 17 mg/kg and 3 mg/kg respectively. Under anesthesia, the mouse’s head was fixed in a custom-made head holder by rigidly clamping between the palate and nasal/frontal bones. The mouth bar was adjusted to align bregma and lambda of the skull in one horizontal plane. Once the mouse’s head was positioned, the scalp was incised along the midline and subcutaneous tissue and muscle were removed to expose the skull. Two holes, 3 and 2 mm in diameter respectively, were drilled to expose the right cerebellum above the cochlear nucleus (5.6–6.5 mm posterior to bregma, 2.1–2.6 mm lateral to the midline) and the left AI (2.2–3.6 mm posterior to bregma, 4–4.5 mm lateral to the midline). After surgery, the animal was placed in a sound-proof chamber to record the response of DCN neurons before and after electrical stimulation of the auditory cortex (Figure 1). During all surgery and electrophysiological experiments, the animal’s body temperature was maintained at a constant 37°C using a feedback-controlled heating pad.

### Acoustic stimulation

Tone bursts, 60-ms duration with 5-ms rise and fall times, were used as acoustic stimuli. They were digitally synthesized and converted into analog sinusoidal waves by an Enhanced Real-time Processor (RP2, Tucker-Davis Tech., Gainesville, FL, USA). The signals were then fed to a tweeter via a digital attenuator (PA5, Tucker-Davis Tech., Gainesville, FL, USA). The output amplitude of the tone bursts was expressed as dB SPL within 1 dB accuracy (reference 20 μPa). The tweeter was placed 45° to the right of and 13 cm away from the mouse’s right ear. During calibration, the tweeter was driven by 20-volt sinusoidal peak-to-peak bursts without attenuation. It was calibrated at the right and left ear of the animal with a condenser microphone (Model 2520, Larson-Davis Laboratories, USA) and a microphone preamplifier (Model 2200C, Larson-Davis Laboratories, USA). Frequencies and intensities of tone bursts were varied either manually or automatically with software (BrainWare, Tucker-Davis Tech., Gainesville, FL, USA).

### Recording and electrical stimulation of the AI

A tungsten electrode (~2 MΩ impedance, FHC, USA) was advanced perpendicular to the surface of the left auditory cortex. The electrode was initially connected to the preamplifier of the data acquisition system. Signals from the electrode were fed to a 16-channel preamplifier, amplified 10,000 times and filtered using a bandwidth of 0.3–10 kHz (RA16, Tucker-Davis Tech., Gainesville, FL, USA), and recorded with software (BrainWare, Tucker-Davis Tech., Gainesville, FL, USA). A pure tone with manual alternation of frequencies and amplitudes was continuously delivered once per second during the electrode penetration. Tone-evoked action potentials were frequently located at layers III/IV of the AI. The lowest amplitude (minimum threshold) and the corresponding frequency at which the neuron showed a response were determined by manual alternation of the tone frequency and amplitude. After 5 ~ 8 penetrations, a rough tonotopy of the AI and the intended location for stimulation of the AI were determined. Once the electrode was in position and the tone-evoked action potentials were observed again, the frequency tunings of the AI neurons were measured with a series of tone bursts at 10 dB above the MT and frequencies that varied from 3 to 40 kHz in 1 kHz steps. Tone stimuli were presented 15 times at each frequency. The frequency to which the neuron showed the largest response magnitude was defined as the BF of the AI neuron. The electrode was then disconnected from the recording system and reconnected to the output of a constant current isolator (A360, WPI Inc., Sarasata, FL) for the ES_AI_. The electrode was advanced to a depth of about 700–800 μm below the brain surface to layer V of the AI where it was maintained for the duration of the experiment. This procedure ensured that the locations of the recording and stimulating sites were in the same AI cell column with the same BFs.

An indifferent electrode was placed on the brain surface just adjacent to the stimulating electrode. The negative pulses (monophasic, 0.1 ms, 500 nA constant current) were generated by a stimulator (Grass S88, Natus Neurology, West Warwick, RI) and a constant-current isolator (A360, WPI, Inc., Sarasata, FL, USA). The electrical pulses were synchronized with the offset of the tone bursts at BF and 20 dB above the MT of the cortical neurons. The combined acoustic from the tweeter and electric stimuli were respectively delivered in both ears (with a right predominance) and to the left AI at a rate of 4 Hz for 7 min; this stimulus paradigm was also used in our previous study (Yan and Ehret, 2002).

### DCN recording

Two tungsten electrodes (~2 MΩ impedance) were dorsoventrally positioned in the right cochlear nucleus. The space between the two electrodes was 100 μm. The location of the DCN was determined physiologically. A pure tone with manual alteration of its frequency and amplitude was continuously delivered once per second during electrode penetration. Tone-evoked responses were commonly observed at a depth of 2.5 mm below the surface of the cerebellum. Once the responses to the tone stimuli were observed, the minimum threshold and the corresponding frequency were measured by manually alternating the frequency and amplitude of the tone. The frequencies at the minimum threshold were measured for each 100-μm interval to map, in an approximate manner, the tonotopic organization along the dorsal-ventral axis. According to the three-dimensional tonotopy of the cochlear nucleus obtained from our previous study (Luo et al., 2009), the frequencies at the minimum threshold decreased dorsoventrally in the same frequency range for both the DCN and posteroventral cochlear nucleus (PVCN) neurons. Thus, the ventral boundary of the DCN was determined when the frequency at the minimum threshold increased as the electrode was advanced into the PVCN. The electrode was then withdrawn in ~100-μm intervals until the action potential recordings stabilized. The minimum threshold and the corresponding frequency of the recorded DCN neuron were determined manually again, and the frequency tunings of the DCN neuron was also measured with the same procedure used for the AI neurons. These response curves of the DCN neurons served as control responses. The frequency to which the neuron showed the largest response magnitude was defined as the BF of the DCN neuron. A negative current of electrical pulses was then delivered to the AI for the micro-electrical stimulation of the AI neurons (4/s for 7 min). The response curves of DCN neurons were again recorded immediately after cortical stimulation and every 30 min until a recovery rate of at least 50% in the BF was obtained.

### Data processing

The tungsten electrode (~2 MΩ impedance) often detected multiunit activities. Cluster cutting isolated and selected single-unit action potentials by examining eight parameters of the action potential waveform, i.e., peak, valley, spike height, spike width, peak time, valley time, and two user-defined voltages (Yan and Ehret, 2002; Yan et al., 2002). Single-unit responses to the series of tones were eventually displayed using post-stimulus time-cumulative (PSTC) histograms with a bin width of 1 ms. The BFs of the DCN neurons were compared before (pre-ES BF) and after (post-ES BF) the ES_AI_. Since the ranges of upward and downward BF shifts were found to be similar, the BF shifts were expressed using a linear kHz scale (Sakai and Suga, 2001; Yan and Ehret, 2002). The changes in the BFs of DCN neurons were analyzed according to the differences in BFs between the recorded DCN neurons and the stimulated cortical neurons.

### Statistical analysis

Data were expressed as mean ± SD. The paired *t*-test (two-tailed) was used to compare the differences between groups of data. A *p*-value of <0.05 was considered to be statistically significant.

## Results

The effects of ES_AI_ were studied in 60 contralateral DCN neurons from 26 mice (2–4 neurons per mouse). The BFs of recorded DCN neurons ranged from 10 to 27 kHz and the BFs of stimulated cortical neurons ranged from 10 to 28 kHz. These values fell within the central range of mouse hearing (Zhang et al., 2005). Our data show that the AI significantly impacts the auditory response of the DCN neurons. The changes in the response properties of DCN neurons occurred within 30 min, peaked at 1.5 h, and recovered at 3 h after the ES_AI_.

**Figure 2:**
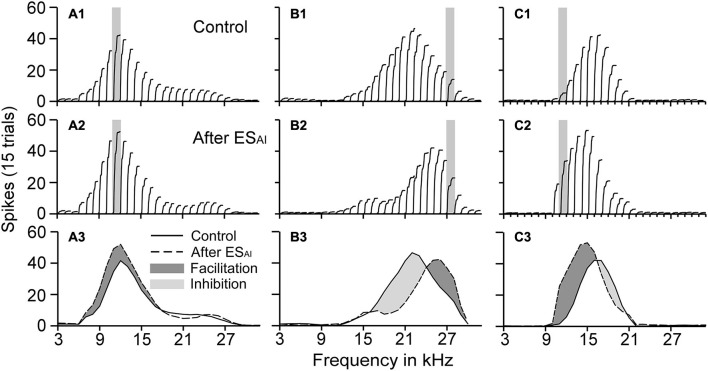
**Three examples illustrating the effects of the ES_AI_ on the frequency tunings of DCN neurons (A–C)**. The ES_AI_ did not change the BF but increased the auditory responses of the matched DCN neurons (A1, A2), while the BFs of the unmatched DCN neurons shifted towards the cortical BF (B1, B2 and C1, C2). The ES_AI_ caused facilitation (A3, B3, C3) and inhibition (B3, C3) of DCN auditory responses. The gray bars in the top two rows of panels represent the BFs of the stimulated cortical neurons. In the bottom row of panels, the dark gray area represents facilitation, whereas the light gray area represents inhibition.

The ES_AI_ clearly changed the frequency tunings of the contralateral DCN neurons. Figure 2 shows three different examples of DCN neurons modulated by ES_AI_. In Figure 2A, the BFs of both cortical and DCN neurons were 11 kHz (matched). The auditory response of the DCN neuron increased after the ES_AI_ (facilitation) while the BF of the DCN neuron did not change. Figure 2B shows that the pre-ES BF of the DCN neuron was 21 kHz while the cortical neuron had a BF of 27 kHz (unmatched). The auditory response at the pre-ES BF (21 kHz, control) of the DCN neuron decreased after the ES_AI_ (inhibition). Hereafter, we use the “facilitation” and “inhibition” for simplicity. These terms represent the overall increase and decrease in neuronal activity without prejudging their underlying neurobiological mechanisms. However, the auditory response at 24 kHz (post-ES BF of DCN neuron) showed the largest increase. Both the facilitation and inhibition by ES_AI_ resulted in a BF shift of the DCN neuron from 21 to 24 kHz. In Figure 2C, the pre-ES BF (16 kHz) of the DCN neuron was higher than the BF (11 kHz) of the cortical neuron (unmatched). The BF of this DCN neuron shifted from 16 to 14 kHz after the ES_AI_. Thus, the changes in the BF of the DCN neurons appear to be determined by the relationship between the BFs of the DCN and AI neurons: (1) the DCN BFs did not change when the BFs of the DCN neurons and those of the AI neurons were matched; and (2) the DCN BFs shifted towards the BFs of the AI neurons when the BFs of the DCN and AI neurons were unmatched.

**Figure 3:**
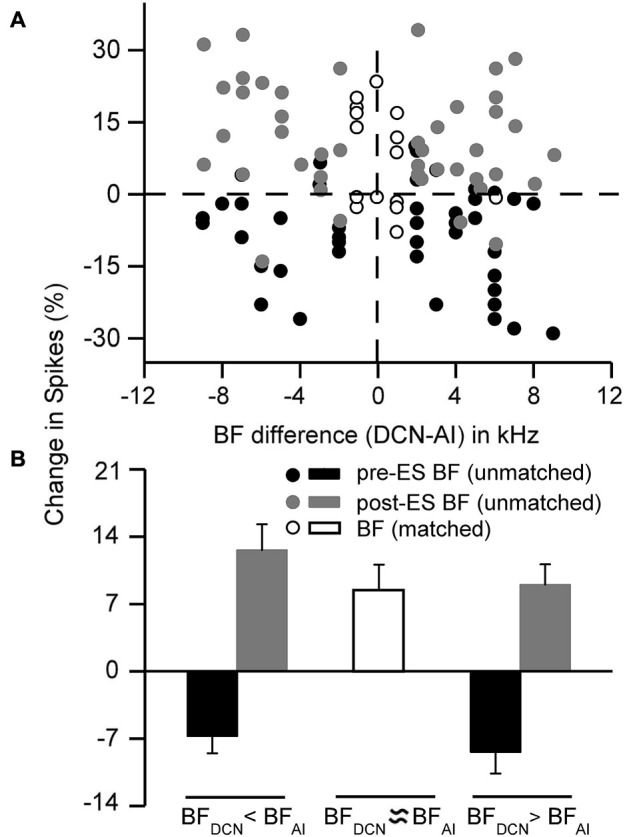
**Frequency-specific changes in the auditory responses (spikes) of DCN neurons after contralateral cortical stimulation**. **(A)** The degree of change was different between the matched and unmatched DCN neurons. Filled black circles indicate responses at pre-ES BFs (Control); filled gray circles indicate responses at post-ES BFs, open circles indicate responses of neurons with matched BF. **(B)** The averaged response change after cortical stimulation. Filled black bars show averaged responses at pre-ES BFs (control); filled gray bars show averaged responses at post-ES BFs, open bar shows averaged response of neurons with matched BF. The error bars represent the SEM.

We also analyzed the effect of ES_AI_ on the frequency tunings of all 60 DCN neurons. The DCN neurons were classified according to the difference between the BFs of the DCN neurons and AI neurons: (1) unmatched group (14 neurons, BF_DCN_ ≈ BF_AI_, Figure 3, open circles and open bar); (2) unmatched Group 1 (21 neurons, BF_DCN_ − BF_AI_ < −1 kHz, Figure 3, filled circles and filled bars); and (3) unmatched Group 2 (25 neurons, BF_DCN_ − BF_AI_ > 1 kHz, Figure 3, filled circles and filled bars). For the auditory response (spikes) at the pre-ES BF and 10 dB above the MT of DCN neurons (control), the response increased after the ES_AI_ for the matched group (Figure 3A, open circles) while it decreased for the unmatched group (Figure 3A, filled black circles). On average, the response of the matched group (Figure 3B, open bar) significantly increased by 9.95% (*p* < 0.01). In contrast, the response of the unmatched group (Figure 3B, filled black bars) significantly decreased by 6.71% (*p* < 0.001) for the unmatched Group 1 and 8.36% (*p* < 0.001) for the unmatched Group 2. For the auditory response (spikes) at the post-ES BF and 10 dB above the MT of DCN neurons, the response increased after ES_AI_ for the unmatched group (Figure 3A, filled gray circles). On average, the response of the unmatched group (Figure 3B, filled gray bars) significantly increased by 12.25% (*p* < 0.001) for the unmatched Group 1 and 10.49% (*p* < 0.001) for the unmatched Group 2. Thus, the ES_AI_ remarkably inhibits the auditory response at the pre-ES BF of DCN neurons while it facilities the auditory response at the post-ES BF when the BFs of the DCN and AI neuron are unmatched.

**Figure 4:**
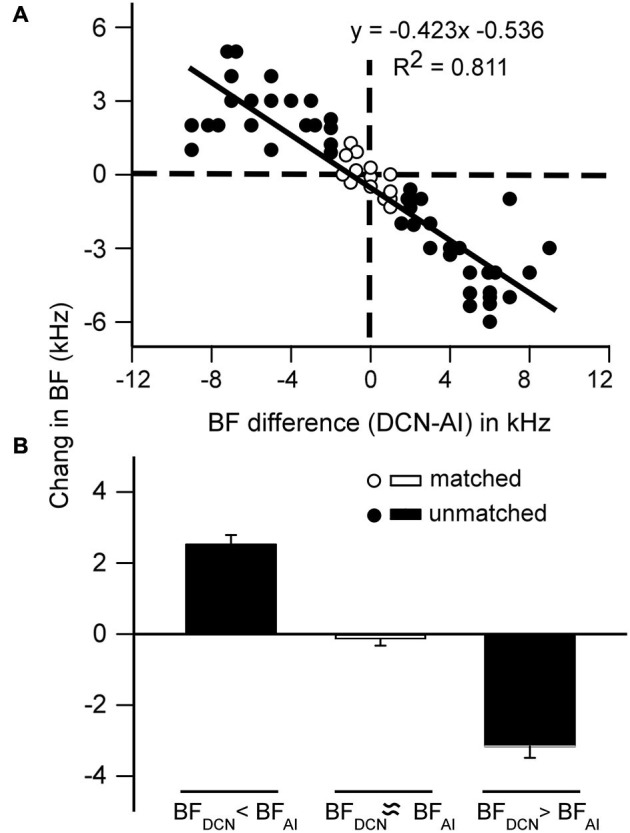
**Frequency-specific changes in the BF of DCN neurons after contralateral cortical stimulation**. **(A)** The degree of change was systematically correlated to the BF difference between the AI and DCN neurons. Open circles, BF shift of neurons with matched BF; filled circles, BF shift of neurons with unmatched BF. **(B)** The averaged BF change after cortical stimulation. Open bar indicates the BF shift of neurons with matched BF; filled bars indicate the BF shift of neurons with unmatched BF. The error bars represent the SEM.

Our results indicate that the shifts in DCN BFs appear to be associated with the BFs of stimulated cortical neurons. To clarify this issue, we further analyzed the correlation of the shifts in DCN BFs to the BFs of the stimulated AI neurons. It became apparent that the BF changes of DCN neurons were systematically associated with the differences in the BFs between the stimulated AI neurons and the recorded DCN neurons (Figure 4A). The shift in BFs was linearly correlated with the differences between the BFs of the AI and DCN neurons (*R*^2^ = 0.811; *p* < 0.01). When the BFs of AI neurons were higher than the BFs of DCN neurons, cortical stimulation significantly increased the BFs of DCN neurons by 2.07 kHz (*p* < 0.001), and when BFs of cortical neurons were lower than the BFs of DCN neurons, cortical stimulation significantly decreased the BFs of DCN neurons by 2.77 kHz (*p* < 0.001) (Figure 4B, filled bars). When the BFs of AI neurons were the same as the BFs of DCN neurons (Figure 4A, open circles), cortical stimulation did not shift the BFs of DCN neurons (Figure 4B, open bar).

## Discussion

Our data clearly demonstrated that the auditory cortex modulated the auditory responses of the DCN neurons in a frequency-specific manner. The BF shifts of DCN neurons were significantly correlated to the differences in the BFs between the stimulated AI neurons and the recorded DCN neurons. Thus, the DCN and AVCN not only exhibit mirror-symmetrical tonotopic maps (Luo et al., 2009), but also share a similar pattern of corticofugal modulation (Luo et al., 2008; Liu et al., 2010). Therefore, the frequency-specificity of corticofugal modulation appears to be dominated by the tonotopy regime of the central auditory system. However, the AVCN and DCN belong to two distinct auditory pathways (lemniscal vs. non-lemniscal, respectively) with different anatomical and physiological properties (Calford and Aitkin, 1983; Imig and Morel, 1983; Hu et al., 1994). Given that the corticofugal modulation of AVCN re-shapes the high-fidelity representation of initial sound information and impacts the sound information that progresses upwards through the lemniscal pathway, what is the function of the corticofugal modulation of the DCN in the non-lemniscal pathway?

The non-lemniscal auditory pathway engages in functions complementary with those of the lemniscal auditory pathway including the integration of information within and across sensory modalities, detection of changes in ongoing stimuli and interestingly, tinnitus (Young et al., 1995; Malmierca et al., 2002; Ryugo et al., 2003; Luo et al., 2012). The hyperactivity of the DCN neurons is considered to be a physiological correlate of the somatosensory tinnitus (Kaltenbach and McCaslin, 1996; Zhang and Kaltenbach, 1998; Baizer et al., 2012), and the corticofugal feedbacks, via the frequency-specific enhancement of the tinnitus-related frequencies, could be partially responsible (Mulders and Robertson, 2009; Eggermont, 2012, 2013). Our findings demonstrate that the DCN is modulated by the AI in a frequency-specific manner, suggesting that the AI may contribute to the chronic form of tinnitus through its modulation of the DCN. The DCN is a relay for auditory information ascending from the periphery to the non-lemniscal pathway. Thus, the AI may also be involved in the other functions of the non-lemniscal pathway.

In addition to tonotopic organization and multisensory inputs, the DCN is a layered structure and consists of distinct cell types including the fusiform cells, giant cells, granule neurons and cartwheel cells (Mugnaini et al., 1980; Browner and Baruch, 1982; Webster and Trune, 1982; Ryugo and Willard, 1985; Willott et al., 1992; Willott, 2001). It has been shown *in vitro* studies that different types of neurons have different membrane properties and firing patterns (Hirsch and Oertel, 1988; Oertel and Wu, 1989; Zhang and Oertel, 1993a,b,c). It would be interesting to determine how the corticofugal modulation is associated with the layered structure and cell types of DCN.

Considering the multiple descending projections within the auditory system, the auditory cortex may modulate the DCN in a direct or indirect manner. The corticofugal projections to the DCN originate from large pyramidal neurons in layer V of the AI, and some fibers directly innervate fusiform cells in all layers of the DCN (Jacomme et al., 2003; Meltzer and Ryugo, 2006; Schofield and Coomes, 2005a,b). These fibers allow the cortical neurons to directly impact the activity of the principal DCN neurons. However, the majority of corticofugal fibers terminate in granule cell lamina between the DCN and the PVCN (Weedman and Ryugo, 1996a,b; Doucet et al., 2002, 2003). The cortical-dependent modulation of DCN neuronal activity through these fibers may involve intrinsic connections between granule cell lamina and the DCN as well as interconnections between the DCN and the ventral cochlear nucleus (Mugnaini et al., 1980; Snyder and Leake, 1988; Wickesberg and Oertel, 1988; Manis, 1989). Although the number of corticofugal fibers to the DCN is much lower than those to the granule cell lamina, we speculate that the highly specific corticofugal modulation is likely mediated by the corticofugal fibers directly projecting to DCN neurons. This is similar to our observations in the case of corticocollicular modulation. The corticocollicular projections mostly target the caudal cortex, dorsal cortex and lateral nucleus of the inferior colliculus, while the tonotopically-organized ICc only receives sparse but tonotopic descending projections from the AI (Saldaña et al., 1996; Winer et al., 1998, 2002; Bajo and Moore, 2005; Bajo et al., 2007). Additionally, direct glutamatergic projections from the AI to the ICc are believed to be responsible for the frequency-specific corticofugal modulation of the ICc (Yan et al., 2005).

In sum, the AI modulates the neural responses in the DCN in a highly frequency-specific manner, similar to the corticofugal modulation of the AVCN. Thus, the frequency-specificity of corticofugal modulation does not appear limited to the lemniscal ascending pathway but dominated by the tonotopy regime of the auditory system. As the DCN receives many descending projections, their involvement in corticofugal modulation requires careful study.

## Conflict of interest statement

The authors declare that the research was conducted in the absence of any commercial or financial relationships that could be construed as a potential conflict of interest.
